# Netrin-4 as a biomarker promotes cell proliferation and invasion in gastric cancer

**DOI:** 10.18632/oncotarget.3400

**Published:** 2015-03-18

**Authors:** Bin Lv, Chunhua Song, Lijun Wu, Qi Zhang, Daisen Hou, Ping Chen, Shunji Yu, Zhicheng Wang, Yiwei Chu, Jun Zhang, Dongqin Yang, Jie Liu

**Affiliations:** ^1^ Department of Digestive Diseases of Huashan Hospital, Fudan University, Shanghai, China; ^2^ Department of Pediatrics, Pennsylvania State University College of Medicine, Hershey, PA, USA; ^3^ Institutes of Biomedical Sciences of Shanghai Medical School, Fudan University, Shanghai, China; ^4^ Department of Laboratory Medicine of Huashan Hospital, Fudan University, Shanghai, China; ^5^ Department of Immunology, Shanghai Medical College, Fudan University, Shanghai, China

**Keywords:** netrin-4, gastric cancer, cell proliferation, cell motility

## Abstract

Gastric cancer (GC) is the second most common cause of cancer-related death with limited serum biomarkers for diagnosis and prognosis. Netrin-4 (Ntn4) is a laminin-related secreted molecule found to regulate tumor progression and metastasis. However, it is completely unknown whether Ntn4 has roles in GC development. Here, we first reported Ntn4 knockdown significantly suppressed cell proliferation and motility, while overexpression or addition of exogenous Ntn4 reversed these effects. In addition, Ntn4 receptor, neogenin (Neo) was also found highly expressed in GC cells and mediated the Ntn4-induced cell proliferation and invasion. Moreover, Ntn4 or Neo silencing decreased the phosphorylation of Stat3, ERK, Akt and p38, indicating multi-oncogenic pathways (Jak/Stat, PI3K/Akt, and ERK/MAPK) were involved in Ntn4-induced effects on the GC cells. Importantly, Ntn4 level was significantly increased in 82 tumor tissues (*p* = 0.001) and 52 serum samples (*p* < 0.0001) from GC patients and positively correlated with Neo expression (*p* = 0.003). Ntn4 expression was negatively correlated with the survival period (*p* = 0.038), and positively associated with the severity of pathological stages of the tumors (*p* = 0.008). Taken together, Ntn4 promoted the proliferation and motility of GC cells which was mediated by its receptor Neo and through further activation of multi-oncogenic pathways. Elevated Ntn4 was detected in both tumor tissues and serum samples of GC patients and suggested a relatively poor survival, indicating Ntn4 may be used as a potential non-invasive biomarker for diagnosis and prognosis of GC.

## INTRODUCTION

Gastric cancer (GC) is one of the most common malignancies worldwide, accounting for 8% of the total cases and 10% of total deaths [1]. Surgical resection remains the first choice for treating GC, although it is always unavailable for a majority of patients due to a delayed diagnosis [2], which is mostly caused by a lack of efficient non-invasive test. Serum biomarkers such as pepsinogen and gastrin-17 contribute little to the diagnosis and prognosis of GC because of poor specificity and sensitivity [3].

Netrins are a conserved family of laminin-like secreted proteins that were originally identified as axonal guidance molecules [4]. Recent studies have demonstrated that netrins are expressed outside the nervous system and involved in a variety of biological processes including tissue morphogenesis [5], angiogenesis [6], lymphangiogenesis [7], tumorigenesis [8], cell migration [9], invasion [10] and adhesion [11], apoptosis [12] and regulation of inflammation [13]. In mammals, netrin system is composed of at least five ligands (netrin 1, 3, 4, G1a, and G1b) and seven potential receptors [deleted in colorectal cancer (DCC), neogenin (Neo), uncoordinated family member 5 (UNC5A-D) and the adenosine A2b receptor (A2b)] [14, 15].

Netrin-4 (Ntn4), the most distant member of netrin family, is described to be a basement membrane component present in the basement membranes of the vasculature, kidney, breast and ovaries [16]. Emerging evidences have indicated that Ntn4 may play an important role in the development of multiple types of cancer. It was found highly expressed in glioblastoma [17] and invasive breast tumors [18, 19]. Also, Ntn4 can act as a regulator of tumor progression and metastasis [8, 17, 20]. However, it is completely unknown whether Ntn4 plays roles in GC tumorigenesis. Furthermore, there's no investigation focusing on the value of Ntn4 as a serum biomarker for cancer diagnosis and prognosis, and the Ntn4 expression in GC patients and its correlation with the clinical features are still undetermined.

Netrin receptors always occupy a major place in netrin signaling transduction. Recently, DCC and UNC5H were reported as netrin-1 (Ntn1) dependent receptor, which ensured cell survival only when Ntn1 was available and induced apoptosis *in vitro* in absence of it [21, 22]. Neo shares 50% amino acid identity with DCC and is widely expressed in a variety of active tissues including gastrointestinal tract, lung, pancreas and developing neurons in the heart [23, 24]. Overexpression of Neo was observed in multiple types of human cancer, especially in aggressive cancers; and unlike DCC and UNC5, Neo enhanced the proliferation and motility of GC cells in an Ntn1 independent manner [24, 25]. Intersetingly, Ntn4 was found to bind to Neo to function in angiogenesis of VEGF-stimulated endothelial cells *in vitro* and *in vivo* [6]. Nevertheless, whether Neo is involved in Ntn4 signailing in GC remains obscure and the underlying mechanism is pooly understood.

Here, we firstly provided evidences for Ntn4 as an oncogene in GC development, and uncover a novel mechanism of it in contribution to GC progression and metastasis through binding to its receptor Neo and futher activating multioncogenic pathways. Importantly, we initially identified the high expression of Ntn4 in both tumor tissues and serum samples of GC patients, which was negatively correlated with survival rate and positively with severity of pathological stages of GC. Our data defined a potential non-invasive biomarker for GC diagnosis and prognosis in clinic.

## RESULTS

### Ntn4 silencing inhibited GC cell proliferation *in vitro*

**Figure 1:**
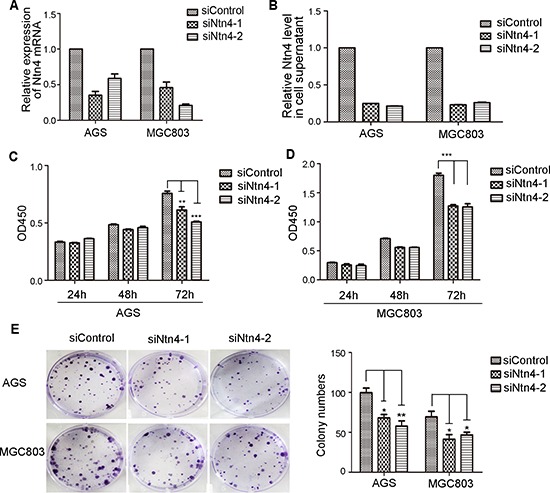
The growth-suppressive effect of Ntn4 knockdown on GC cells **A–B.** Ntn4 was efficiently knockdown by two independent Ntn4 siRNAs in AGS and MGC803 cells. Ntn4 expression was examined by Q-PCR (A) and ELISA (B) after transfection for 48 hours. **C–D.** Ntn4 ablation suppressed the proliferation of gastric cancer cells by CCK8 assays. The siRNAs were transfected into AGS (C) and MGC803 (D) cells as indicated for 24–72 hours. **E.** Ntn4 silencing reduced colony formation in AGS and MGC803 cells. Data are presented as mean ± SEM from three independent experiments with each running in triplicate. Unpaired student's *t*-test was used for the comparison between the two groups (*n* = 3, *p < 0.05, **p < 0.01, ***p < 0.001).

To address the efficacy of Ntn4 on GC cells, we knocked down Ntn4 in AGS and MGC803 cells using two siRNA oligoes targeting two well-identified regions of Ntn4 sequences (named siNtn4-1, siNtn4-2). As shown in Fig. 1, each GC cell line transfected with Ntn4 siRNA showed efficient silencing of Ntn4 expression, as determined by real-time RT-PCR and ELISA (Fig. 1A–1B). We observed the effect of Ntn4 knockdown on the proliferation of the GC cells. With CCK8 assay, we found that siNtn4 significantly decreased the viability of cells compared with scrambled siRNA (siControl) (Fig. 1C–1D). We also examined the effect of siNtn4 by clonogenic assay and found that Ntn4 knockdown notably suppressed clonogenic survival by inhibiting colony formation as shown in Fig. 1E. Taken together, our results demonstrated that siNtn4 inhibited cell proliferation of the GC cells.

### Ntn4 silencing diminished the GC cell motility

**Figure 2:**
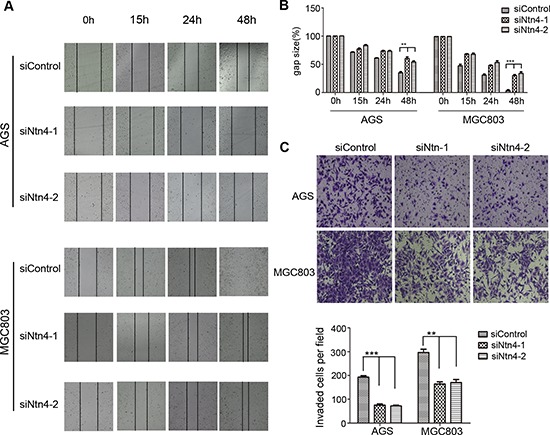
Ntn4 silencing suppressed the migration and invasion of GC cells **A–B.** Ntn4 knockdown slowed the wound healing in AGS and MGC803 cells. The monolayer of cells transfected with siControl or siNtn4-1/2 was disrupted with a tip, the cells migrated into the disrupted gap of the monolayer were observed under microscope and photographed at 0, 15, 24, and 48 hours; Magnification, × 100 (A); the gap size was measured and plotted as the percentage of the original time point (0 hour) (B). **C.** Ntn4 knockdown interrupted cell invasion. siControl or siNtn4 cells were plated into a matrigel-coated 8 μm tranwell. After 24 hours incubation, invaded cells on the bottom surface were fixed and counted after staining with crystal violet; Magnification, × 100. Mean ± SEM, *n* ≥ 3. **p < 0.01, ***p < 0.001.

Previous studies have reported that Ntn4 was involved in cell apoptosis and motility. However, cell cycle profile of the Ntn4-silenced GC cells by PI staining revealed no obvious cell cycle arrest or apoptosis (Suppl. Fig. 1). We next investigated whether Ntn4 knockdown could regulate cell migration and invasion. The wound healing assay was performed to examine the migration of GC cells treated with siNtn4 or siControl. Microscopic examination at 15, 24 and 48 h showed that Ntn4 knockdown significantly inhibited migration in AGS cells (Fig. 2A, upper panels) as well as in MGC803 cells (Fig. 2A, lower panels), as shown by a delayed wound closure in siNtn4 cells compared with control cells. At 48 h, the gap sizes of siControl and siNtn4-1 in AGS were 35% vs 60%, while in MGC803 were 3% vs 34% (Fig. 2B). To further validate this observation, a matrigel invasion assay in transwell culture chambers was performed to evaluate the invasive potentials of GC cell lines. As shown in Fig. 2C, the number of AGS cells transfected with siNtn4-1/2 that passed through matrigel was only 39%/37% as compared with cells transfected with siControl. Similar results of invasion assay were obtained in MGC803 cells (Fig. 2C). These findings indicated that Ntn4 silence attenuated the migration and invasion of GC cells.

### Ntn4 promoted cell proliferation and cell motility in GC cells

**Figure 3:**
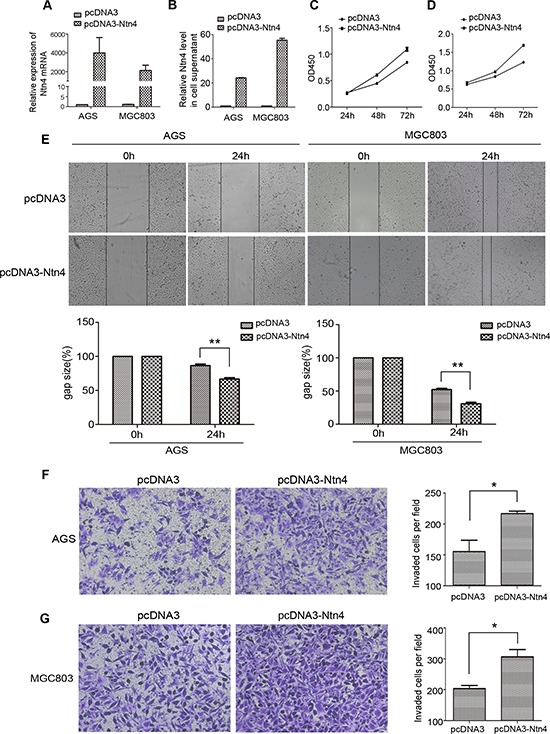
Overexpression of Ntn4 promoted the growth, migration and invasion of GC cells **A–B.** The overexpression of Ntn4 was achieved in AGS and MGC cells by transient transfection of pcDNA3-Ntn4 for 48 hours and with pcDNA3 vector (pcDNA3) only as control. Expression of Ntn4 was measured with Q-PCR (A) and ELISA (B). **C–D.** The enhanced expression of Ntn4 accelerated the growth of AGS (C) and MGC803 (D) cells. The cell proliferation assay was performed with CCK8 assay at 24, 48 and 72 hours, respectively after the transfection with pcDNA3 or pcDNA3-Ntn4. **E.** The enhanced expression of Ntn4 boosted the wound healing of AGS and MGC803 cells. The monolayer of cells transfected with pcDNA3 or pcDNA3-Ntn4 was disrupted with a tip and photographed under microscope at 0 and 24 hours; Magnification, × 100. **F–G.** The enhanced expression of Ntn4 promoted the invasion of AGS (F) and MGC803 (G) cells. Cells were transfected with pcDNA3 or pcDNA3-Ntn4 for 48 hours and subjected to the invasion assay; Magnification, × 100. Mean ± SEM, *n* ≥ 3. **p < 0.01.

To further assess the role of Ntn4 in the proliferation and migration of GC cells, pcDNA3-Ntn4 or control vector (pcDNA3) was transiently transfected into AGS and MGC803 cells. Real-time RT-PCR and ELISA were performed to confirm Ntn4 gene overexpression at the mRNA level (Fig. 3A) and protein level (Fig. 3B). CCK8 assay revealed that the overexpression of Ntn4 obviously enhanced cell proliferation in both AGS and MGC803 cells (Fig. 3C–3D). Moreover, wound-healing assay showed that the gap sizes of AGS and MGC803 cells with pcDNA3-Ntn4 transfection were significantly decreased by 20% and 21%, respectively, at 24 h compared to that with pcDNA3 transfection (Fig. 3E). The invasion assay showed that Ntn4 overexpression was associated with an increase in invaded cells in the pcDNA3-Ntn4 group compared with control cells (Fig. 3F–3G). These data supported Ntn4 overexpression promoted cell growth, migration and invasion of GC cells, further confirming the role of Ntn4 in regulation of cell proliferation and motility.

Previous studies revealed that the effects of Ntn4 on glioblastoma cells are concentration dependent [17]. Therefore, we assessed the cell invasion of AGS and MGC803 cells with increasing doses of exogenous Ntn4. The result showed that exogenous Ntn4 promoted cell invasion with a maximum at 50 ng/ml (Suppl. Fig. 2), implying that the effects of Ntn4 on invasion of the GC cells were also dose-dependent as reported in glioblastoma cells.

### Silencing of Ntn4 receptor, neogenin also suppressed the proliferation and invasion of GC cells

**Figure 4:**
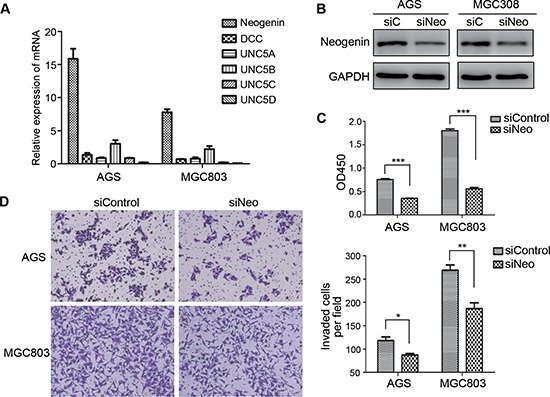
Down regulation of neogenin inhibited cell growth and invasion **A.** The expression of Ntn4 receptors, neogenin (Neo), DCC and UNC5A-D were detected by Q-PCR. **B.** Neo expression was detected in the cells by western blot at 48 hours after transfected with siNeo or siControl. **C.** Neo silencing suppressed cell proliferation. Cells were transfected with siNeo or siControl for 48 hours and subjected to CCK8 assay. **D.** Neo knockdown restrained cell invasion. Cells were transfected with siNeo or siControl for 48 hours and subjected to the invasion assay; Magnification, × 100. Mean ± SEM, *n* ≥ 3. *p < 0.05, **p < 0.01, ***p < 0.001.

Ntn4 is a secretory protein and exerts its effects by binding to its receptor on cell membrane. To identify which receptors that mediate the biologic functions of Ntn4 in GC cells, we first determined the expression of the known receptors of Ntn family including Neo, DCC and UNC5A-D in the AGS and MGC803 cells by Real-time PCR, and found that Neo is the highest expressed receptor of Ntn4 in these two GC cells (Fig. 4A). Therefore, we hypothesized that Neo may be invovled in Ntn4 induced cell proliferation and motility changes.

To further address the role of Neo in the growth of GC cells, we knocked down Neo (named siNeo) in AGS and MGC803 cells and the western blot showed that the siRNA efficiently reduced the expression of Neo in the two cell lines (Fig. 4B). The CCK8 assay showed that siNeo significantly decreased the growth of AGS and MGC803 cells by 32% and 54%, respectively, at 72 h in comparison to siControl (Fig. 4C). In addition, fewer cells treated with siNeo passed through the matrigel than that with siConrol (Fig. 4D, left panel), with the number of AGS 87/118 and of MGC803 187/269 (Fig. 4D, right panel). These findings showed that silence of Ntn4 receptor, Neo could also result in the cell proliferation arrest and reduction of the cell invasion in both GC cell lines, which suggested the effect of Ntn4 on cell proliferation and motility may be mediated through its receptor, Neo.

### Neo mediated the effect of Ntn4 on cell proliferation and invasion

**Figure 5:**
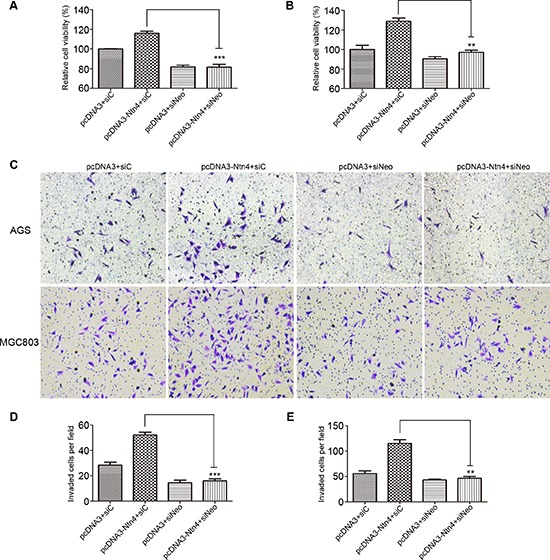
Neo knockdown reversed the promoted effects of Ntn4 on cell proliferation and invasion **A–B.** Ntn4 overexpression promoted cell growth in a Neo dependent manner. The cell proliferation was performed in AGS (A) and MGC803 (B) cells co-transfected with pcDNA3+siC, pcDNA3+siNeo, pcDNA3-Ntn4+siC or pcDNA3-Ntn4+siNeo for 48 hours with CCK8 assay. **C–E.** Ntn4 overexpression couldn't increase cell invasion in the absence of Neo. Cells were co-transfected with pcDNA3+siC, pcDNA3+siNeo, pcDNA3-Ntn4+siC or pcDNA3-Ntn4+siNeo for 48 hours and subjected to the invasion assay (C); Magnification, × 100. The invaded AGS (D) or MGC803 (E) cells on the bottom surface were counted and analyzed. Mean ± SEM, *n* ≥ 3. **p < 0.01, ***p < 0.001.

Next, we explored the influence of Neo ablation on Ntn4-induced cell proliferation and invasion in the GC cells. As we observed in Fig. 5A–5B, the AGS and MGC803 cells treated with pcDNA3-Ntn4 grew faster than that with pcDNA3, wheras Neo depletion could partially reverse the cell growth caused by Ntn4 overexpression. Furthermore, we found that Neo knockdown blocked the Ntn4 overexpression-induced invasion of AGS and MGC803 cells (Fig. 5C–5E). These data further confirmed that Ntn4 accelerated cell proliferation and invasion only in the presence of its receptor, Neo.

### Effects of Ntn4 on cell invasion-related biomarkers

**Figure 6:**
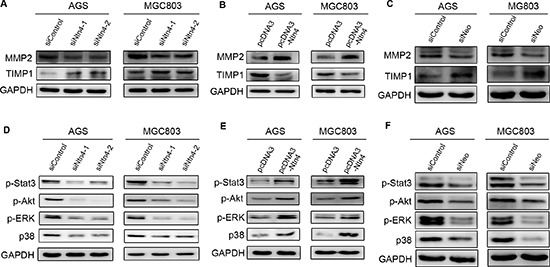
Effects of Ntn4 or Neo on JAK/Stat, PI3K/Akt, ERK/MAPK signaling **A–B.** Ntn4 silencing down-regulated the expression of MMP2 (A) and up-regulated the expression of TIMP1 (B). Cells were transfected with siRNAs for 48 hours and subjected to western blot with indicated antibodies. **C.** Neo ablation down-regulated MMP2 and up-regulated TIMP1. **D–E.** Effect of Ntn4 knockdown (D) and Ntn4 overexpression (E) on phosphorylation of Stat3, Akt, ERk and p38. The phosphorylation level was evaluated by western blot with specific antibodies to p-Stat3, p-Akt, p-ERK and p-p38, respectively. **F.** Effect of Neo knockdown on p-Stat3, p-Akt, p-ERK and p-p38.

In order to further understand how Ntn4 induced the cell invasion, we examined the expression of two invasion-related biomarkers, matrix metalloproteinase 2 (MMP2) and tissue inhibitor of metalloproteinase 1 (TIMP1). As shown in Fig. 6A and 6C, when blocking the Ntn4-Neo complex through interfering either Ntn4 or Neo, we observed the expression of MMP2 was significantly downregulated, meanwhile TIMP1 was upregulated as compared with control. Conversely, overexpression of Ntn4 increased MMP2 level and decreased TIMP1 level, implying that Ntn4 could enhance invasion of GC cells by leading to an imbalance between MMP2 and TIMP1 (Fig. 6B).

### Effects of Ntn4 on JAK/Stat, PI3K/Akt, and ERK/MAPK signaling pathways

In order to gain insight into the molecular mechanism underlying the Ntn4 induced expression changes of invasion-related biomarkers, we examined the effect of Ntn4 on oncogenic signaling pathways. We found that Stat3 phosphorylation was reduced in AGS and MGC803 cells with Ntn4 knockdown for 48 h compared to that with siControl; also, the decreased phosphorylation of ERK, Akt and p38 was observed in the cells (Fig. 6D). Conversely, the phosphorylation of Stat3, ERK, Akt and p38 was increased in cells with Ntn4 overexpression (Fig. 6E).

Furthermore, we observed that Neo knockdown decreased the phosphorylation of Stat3, Akt, ERK and p38 in the AGS and MGC803 cells (Fig. 6F). Therefore, our results declared that the activation of JAK/Stat-PI3K/Akt-ERK/MAPK axis was essential for Ntn4-promoted cell proliferation and cell motility.

### Ntn4 overexpression was observed in GCs and predicted a poor prognosis in GC patients

To investigate the clinical significance of Ntn4 in GCs, we first determined the expression of Ntn4 in 82 GC patients' samples.

**Figure 7:**
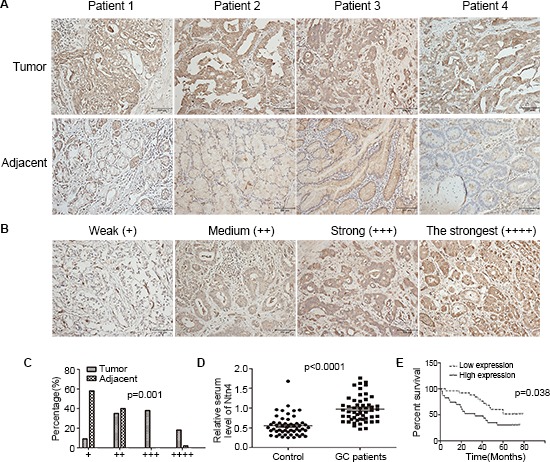
Ntn4 was overexpressed in GCs and negatively correlated with patient survival **A.** IHC staining of human GC tissues using Ntn4-specific antibody, as described in Methods; Magnification, × 200. **B–C.** Classification of samples according to the intensity of staining of Ntn4 expression (*n* = 82, *p* = 0.001). **D.** ELISA analysis to determine serum level of Ntn4 in GC patients and normal controls (*n* = 52, *p* < 0.001). **E.** Kaplan-Meier curves for GC patients' overall survival in the patients with high Ntn4 expression and low Ntn4 expression (*n* = 48, *p* = 0.038).

As shown in Fig. 7A, Ntn4 expression was significantly higher in the tumor tissues (up panels) compared with adjacent tissues (down panels). Based upon the intensity of staining, we classified the samples into four groups with increasing staining intensity from weak (+) to the strongest (++++) (Fig. 7B). Its expression in majority of GC tissues was high, falling into group 3 and 4 and the expression in adjacent tissues was weak, falling into group 1 and 2 (Fig. 7C, *p* = 0.001). We further quantified the protein level of Ntn4 in GC patient's serum, as compared with normal population by ELISA analysis. As shown in Fig. 7D, the levels of Ntn4 in the sera of the tumor patients were significantly higher than those of the normal samples (*n* = 52, *p* < 0.0001).

Next, we analyzed the correlation between Ntn4 expression and patients' overall survival. Kaplan–Meier analysis showed that the patients with high Ntn4 expression (+++, ++++) have significant lower overall survival rate compared with those with low Ntn4 expression (+, ++) (Fig. 7E, *p* = 0.038).

In addition, we analyzed the Ntn4 expression with clinicopathological stages. The patients' information was shown in Suppl. Table 1. The clinicopathological stages in the patients were classified based on TNM system with the AJCC criteria; and 13 patients (16%) were in stage I, 24 patients (29%) in stage II, 39 patients (48%) in stage III and 6 patients (7%) in stage IV. In the patients with stage I and II, Ntn4 was significantly up-regulated in tumor tissues compared with the adjacent tissues (Suppl. Fig. 3). Moreover, the high Ntn4 expression was detected in 71% (34/45) tumor samples with stage III and stage IV but only 12% (12/37) with Stage I and II. Statistical analysis showed that Ntn4 expression was positively correlated with severity of pathologic stages (*p* = 0.008).

Finally, we evaluated the correlation of Ntn4 expression with Neo. As shown in Suppl. Fig. 4, the Neo expression was also classified into four groups with increasing staining intensity from weak (+) to the strongest (++++) in tumor tissues. By using Fisher's exact test, we found that Ntn4 expression was positively associated with Neo expression (Table 1, *p* = 0.003), indicating that high Neo expression may play a key role in Ntn4-induced tumorigenesis in the GC.

**Table 1:** Relationship between Ntn4 and Neo expression in 36 tumor tissues

Ntn4 expression		Neo expression		
		Weak	Strong	*P* value
Weak	16	13	3	0.003
Strong	20	6	14	

Taken together, these findings demonstrated that Ntn4 overexpression was involved in GC tumorigenesis or the maintenance of its malignant phenotypes. It also indicated Ntn4 overexpression has a potential to be a non-invasive biomarker for diagnosis and prognosis of GC.

## DISCUSSION

In this study, we first reported the oncogenic effects of Ntn4 on GC development and identified a novel molecular mechanism. More importantly, for the first time we provided evidences for Ntn4 as a non-invasive biomarker for diagnosis and prognosis of GC.

Originally identified as axonal guidance molecules, netrin family are recently reported to be involved in tumorigenesis. Nertin-1 (Ntn1), structurally related to the γ-chain of laminins, is the first identified member of netrin family and its overexpression has been observed in many human cancers including those of breast, pancrease, cervix, colon, medulloblastoma and rectum [26–29]. Previous studies have demonstrated Ntn1 promotes cell invasiveness, angiogenesis and cancer progression [10, 13, 30]. Ntn4, a more distantly related netrin, displays homology to the β-chain of laminins. Like Ntn1, Ntn4 is able to stimulate tumor proliferation and angiogenesis [6, 17, 20]. These data, along with the broader Ntn1 studies, suggest an essential role for Ntn4 in controlling tumor growth and development. Here, we extended this work to gastric cancer and discovered Ntn4 was highly expressed in GC tissues. In GC cells, knockdown of Ntn4 significantly suppressed cell proliferation and cell motility, while overexpression of Ntn4 or addition of exogenous Ntn4 obviously promoted cell proliferation and cell motility, implying the oncogenic effect of Ntn4 in GC for the first time. Importantly, we initially found that Ntn4 level was significantly increased in serum samples from GC patients, which supported its diagnostic value in GC detection. Furthermore, the elevated Ntn4 was negatively correlated with the survival period and positively associated with the severity of pathological stages in GC patients, indicating Ntn4 can also be applied for GC prognosis.

Ntn4 is a secreted protein, which binds to its receptor to exert its effects. Howewver, the receptors and downstream signaling pathway of Ntn4 mediating these functions are not clear. In contrast to other netrins receptors as DCC and UNC5C, Neo is widely expressed in a variety of active tissues including gastrointestinal tract, lung, pancreas and developing neurons in the heart [23]. Its overexpression is also thought to be associated with aggressive cancers [24, 25]. Recent studies have reported that Ntn4 could bind to Neo to modulate tumor angiogenesis [6, 8]. Here, we observed that Neo was highly expressed in AGS and MGC803 cells as well as clinical GC tissues, and found that Neo ablation could block the cell proliferation and motility. These data indicated that Neo itself may be also involved in the tumorigenesis by promoting cell proliferation and invasion in GC. Moreover, Neo knockdown could efficiently reverse the cell proliferation and invasion promoted by Ntn4 overexpression, suggesting Ntn4-induced cell proliferation and motility is mediated by Neo at least partly if not all in the cells. Interestingly, we discovered a significant positive correlation between Ntn4 and Neo expression in clinical tissue samples, which strongly supported our hypothesis that Ntn4 regulates cell growth and invasion by interacting with its receptor Neo.

Functions described for netrins include the regulation of cell migration, axon extension and guidance, cell-cell and cell-substrate adhesion, cell survival and cellular differentiation. Netrins family members exert their functions by activation of intracellular signaling pathways. Studies investigating the signal transduction mechanisms engaged by secreted netrins have focused largely on Ntn1 and relatively little is known about the specific signaling mechanisms activated by other netrin family members [31]. It is reported that Ntn4 induces proliferation, migration and survival of lymphatic endothelial cells by stimulating phosphorylation of ERK1/2 and Akt, and also through activation of mTOR signaling pathways [7]. Consistently, we found that Ntn4 overexpression increased the phosphorylation of ERK and Akt, while Ntn4 and neo knockdown decreased their phosphorylation. Furthermore, we discovered that Stat3 and p38 also participated in Ntn4-induced cell proliferation, migration and invasion as envidenced by an increase of phosphorylation modification of them. These findings declared that Ntn4 may promote cell proliferation and motility by activation of JAK/Stat3, Akt/PI3K and ERK/MAPK signaling pathways in GC cells.

In order to further understand the molecular mechanism that Ntn4 induces the cell invasion, we also observed the effect of Ntn4 and Neo on expression of MMP2 and TIMP1, two key molecules for cell invasion, and found the Ntn4- and Neo-dependent increase of MMP2 and decrease of TIMP1. It is reported that activation of both PI3K/Akt and ERK/MAPK pathways increases MMP2 and decreases TIMP1 expression in cancer cells [32–35]. We considered that Ntn4 overexpression causes such changes of MMP2 and TIMP1 expression and therefore enhanced cell invasion through activating PI3K/Akt and ERK/MAPK pathways. However, how Ntn4 causes such changes in MMP2 and TIMP1 expression through activation of these pathways needs to be further clarified.

In summary, our study showed that Ntn4 promotes the proliferation and motility of GC cells in an Neo–dependent manner through further activation of multi-oncogenic pathways. Moreover, Ntn4 level was dramatically up-regulated in both tumor tissues and serum samples of GC patients, and the high expression of Ntn4 was significantly correlated with a short survival time and a high TNM stage, indicating it may be used as a potential non-invasive biomarker for diagnosis and prognosis of GC.

## METHODS

### Cell culture and reagents

Human gastric cancer cell lines AGS and MGC803 were obtained from the Cell Bank of Chinese Academy of Medical Science (Shanghai, China). These cells were cultured in Dulbecco's modified Eagle's medium containing 10% fetal bovine serum (Invitrogen Life Technology, Carlsbad, CA), penicilin (100 U/mL), and streptomycin (100 mg/mL). Recombinant Nnt4 was purchased from R&D Systems, Minneapolis, MN.

### Patient samples

Gastric cancer paraffin-embedded tissues were obtained from Department of Pathology, Huashan Hospital. Briefly, samples from 82 gastric cancer patients, who received surgical treatment at Huashan Hospital in 2005, were collected and confirmed as gastric adenocarcinoma, and then made available for this study. Follow-ups were terminated until July 2011. During the follow-up period, a total of 34 patients were lost, which meant 48 patients were eventually available at the final follow-up, giving a follow-up rate of 58.5%.

Serum samples were from gastric cancer patients who had not received any radiotherapy or chemotherapy before surgical resection at Huashan hospital in 2014. All of them were collected just before surgery and confirmed as gastric adenocarcinoma after postoperative pathologic examination. The control patients were noncancerous age- and sex-matched volunteers who may have different diseases.

The use of all tissue blocks and serum samples for this study was approved by the Institutional Ethics Review Board of Huashan Hospital.

### Immunohistochemical staining

Immunohistochemical (IHC) staining was performed using Dako EnVision kit (Dako, Denmark), and detected by Dako Liquid DAB. The tissue slides were heated by microwave (low, medium and high fire, 5 minutes each) for antigen retrieval with citrate buffer (pH 6.0) as the solution. Then were incubated with anti-Ntn4 antibodies (diluted 1:200; R&D Systems) or anti-Neo antibodies (diluted 1:100; Santa Cruz Biotechnology, Santa Cruz, CA) at 37°C for 60 minutes, and then incubated overnight at 4°C in moist chambers. The Ntn4 primary antibody was detected using the ImmPRESS™ Reagent Kit (Vector, Burlingame, CA). The Neo primary antibody was detected using the Dako EnVision™ Kit. Reaction products were visualized by Dako Liquid DAB Substrate-Chromagen System and then counterstained with haematoxylin. Negative controls were samples that were incubated with normal goat or mouse serum. All immunostanined sections were determined respectively by two pathologists who did not know the research contents. Ntn4 expression was divided into four groups based on staining intensity: + ~ + + + +. + ~ + + represent low expression and + + + ~ + + + + represent high expression.

### Quantification of cell supernatant and serum Nnt4 by ELISA

One hundred microliters of cell supernatant or serum was used for the Ntn4 assay, which used an ELISA kit (Enzo Life Science Inc, USA). Briefly, a total of 100 ul/well serum and standard samples were added to antibody-coated 96-well plates and incubated for 2 hours at room temperature, followed by addition of biotin-conjugated polyclonal antibody specific for Ntn4 and incubation for 1 hour. And then washed and incubated with avidin conjugated to HRP (eBioscience, San Diego, CA) for 1 hour. Color was developed using TMB substrate (eBioscience), stoped by adding sulfuric acid and measured using a plate reader (M200 Pro, Tecan) at a wavelength of 450 nm. The assay was linear from 10 to 2000 pg.

### Cells transfection and overexpression

Cells were transfected with siRNA or plasmid vectors using Lipofectamine RNAiMax or Lipofectamine 2000 (Invitrogen) according to the manufacture's instruction. The sequences of siRNAs are as follows: siNtn4-1: 5′GUCCAUGGGAAGUGUAUGUTT 3′; siNtn4-2: 5′CUCACCUAAUUGUGAUG UUTT 3′; siNeo: 5′CATTACCTCCCACTTCACT 3′ [36]. The siRNAs were from GenePharma (Shanghai, China). The pcDNA3 control vector and pcDNA3-Ntn4 expression vector were kindly offered by Marko Hyytiäinen [17].

### Quantification of Nnt4 and its receptors by Real-time RT-PCR

Total cellular RNA was isolated using Trizol (Invitrogen). Reverse transcription was carried out with PrimeScript™ RT Master Mix (TaKaRa, Japan) according to the manufacturer's instructions. The cDNA was amplified by Power SYBR Green PCR Master Mix (Applied Biosystems, Foster City, USA) according to the manufacturer's protocol in an Applied Biosystems 7500 sequence detection system. Levels of gene expression were determined by ΔΔCT method, with the results being expressed as mRNA expression levels normalized to the levels of GAPDH.

### Wound healing assay and matrigel invasion assay

For wound healing assay, cells were treated siRNA or plasmids as mentioned above. After 48 hours of culture, the confluent monolayers were wounded using a sterile pipette tip and evaluated under phase contrast microscopy at 0–48 h using microscope (Leica, Wetzlar, Germany). The migration rate was analyzed with the ImageJ program.

For the invasion assay, matrigel solution (BD Biosciences) was prepared in serum-free cell culture medium at a dilution of 1:8, coated with the 24-well transwell chambers (Corning Costar, USA) overnight at 37°C before cell seeding. The cells were cultured in the chamber with serum-free media containing 1% BSA in triplicate at 1 × 10^5^ cells per well. After 24 hours of cultivation, the insert were removed by wiping with a cotton swab. Cells that migrated to the bottom surface of the insert were fixed with 4% paraformaldehyde, stained with 0.4% crystal violet, and counted in five random fields.

### Western blotting analysis

Cell lysates were extracted with cell lysis buffer (Beyotime, Hangzhou, China) and the protein concentration was quantified using an Enhanced BCA Protein Assay Kit (Beyotime). The primary antibodies used were as follows: anti-MMP2, anti-TIMP1, anti-GAPDH (Epitomics, Hangzhou, China); anti-neogenin (Santa Cruz Biotechnology), anti-netrin 4 (R&D Systems); anti-phosphorylated Stat3, anti-phosphorylated Akt, anti-phosphorylated ERK, anti-phosphorylated p38 (Cell Signaling Technology, Danvers, MA, USA).

### Cell proliferation and clonogenic assay

Cells transfected with the indicated siRNAs or plasmids were seeded into 96-well plates 2000 cells per well in triplicate for CCK8 colorimetric assay (Dijindo, Japan) according to the manufacture's specifications. For the clonogenic assay, the split cells were seeded into six-well plates and cultured for 10 days. The colonies on the plates were fixed with 4% paraformaldehyde, stained with crystal violet and counted.

### Statistical analysis

The statistical significance of differences between groups was assessed using the GraphPad Prism 5 and SPSS 16.0 softwares. The unpaired two-tailed t-test was used for the comparison of parameters between two groups. Survival curves were plotted by the Kaplan–Meier method and statistical differences were analyzed using the log-rank test. Correlation between Ntn4 expression and clinicopathological was performed by the two tailed Mann–Whitney *U*-test or Kruskal–Wallis test. Fisher's exact test was used for testing relationship between Ntn4 and Neo expression. For all the tests, three levels of significance (*p* < 0.05, *p* < 0.01 and *p* < 0.001) were used.

## SUPPLEMENTARY FIGURES AND TABLE


