# Supplementary material for: Netrin-4 as a biomarker promotes cell proliferation and invasion in gastric cancer
Source: Oncotarget. 2015 Mar 18;6(12):9794–806. doi: 10.18632/oncotarget.3400 (PMC4496398; doi:10.18632/oncotarget.3400)
Supplement: Supplementary file 1 [file oncotarget-06-9794-s001.pdf]

SUPPLEMENTARY FIGURES AND TABLE

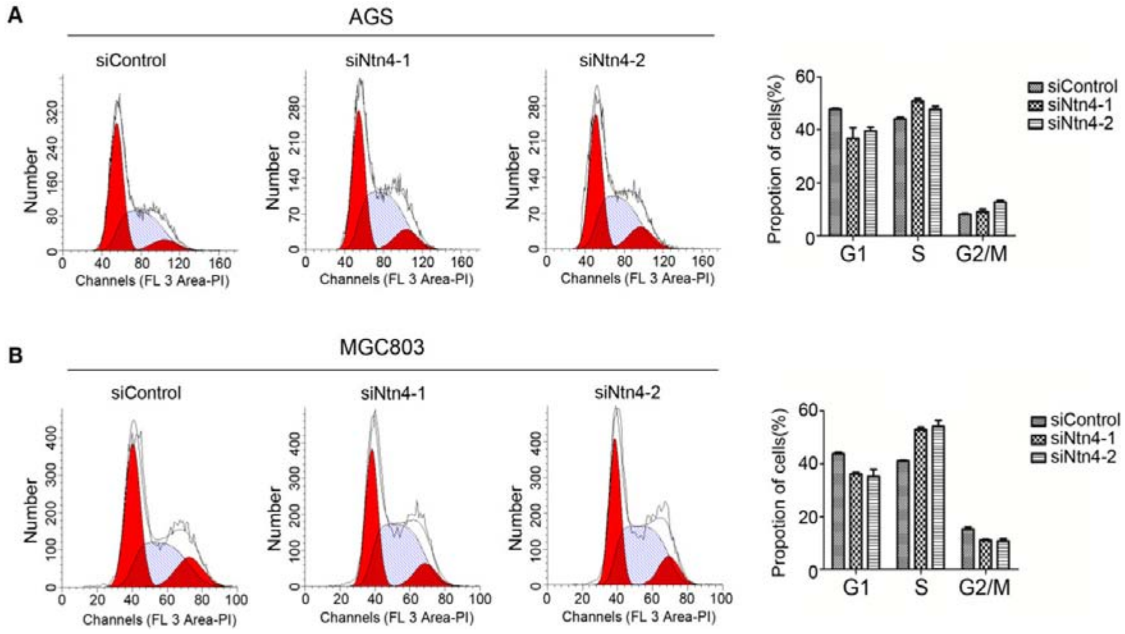

Supplementary Figure S1: Ntn4 silencing had little effect on cell cycle. AGS **(A)** and MGC803 **(B)** were transfected with siControl or siNtn4-1/2 for 48 hours and subjected to PI staining and FACS analysis to determine cell cycle profile.

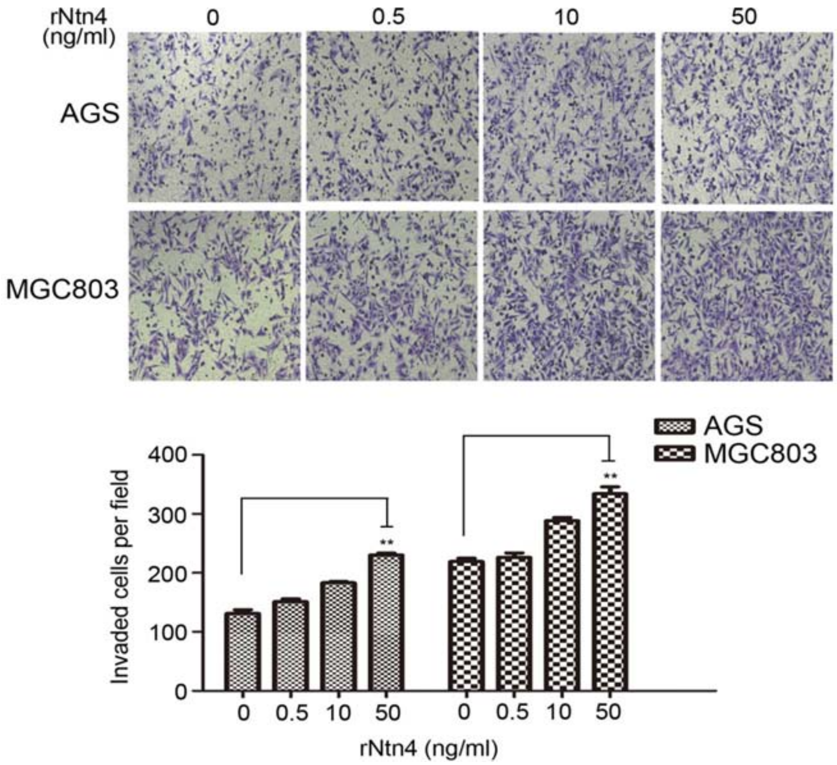

Supplementary Figure S2: Recombinant Ntn4 promoted cell invasion in a dose-dependent manner. AGS and MGC803 cells were cultured in the presence of recombinant Ntn4 with the indicated concentrations for 48 hours and then subjected to invasion assay. Magnification, × 100. Mean ± SEM, $n \geq 3$. $**p < 0.01$.

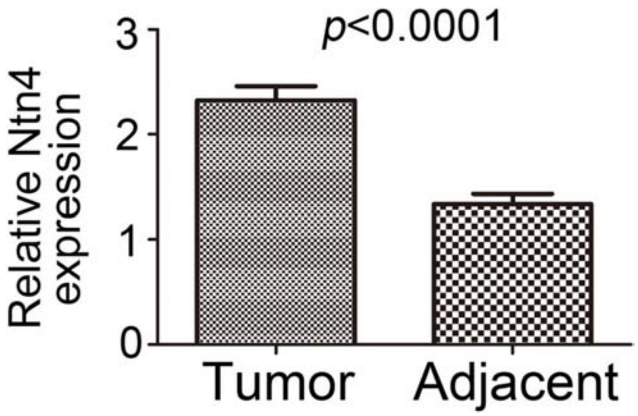

Supplementary Figure S3: Ntn4 was significantly up-regulated in GC tumor tissues from the patients with the early stage. In the patients with the early stage of GC which means TNM I and II stage, Ntn4 was significantly up-regulated in tumor tissues compared with the adjacent tissues by IHC staining. Mean ± SEM. $n = 37$, $p < 0.001$.

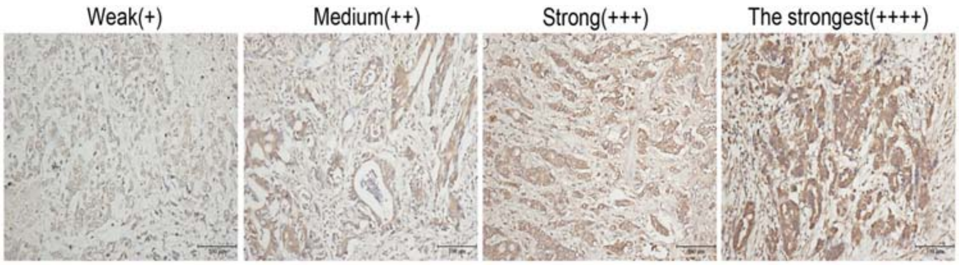

Supplementary Figure S4: Classification of samples according to the intensity of staining of Neo expression in GCs. Magnification, × 200. (n = 36).

Supplementary Table S1. Clinicopathologic correlation of Ntn4 expression in 82 tumor tissues from gastric cancer patients

		Ntn4				
**Characteristic**	**n**	**I**	**II**	**III**	**IV**	***p* Value**
Sex						0.132
Male	56	7	17	20	12	
Female	26	0	12	11	3	
Age(years)						0.209
<= 55	25	4	10	9	2	
> 55	57	3	19	22	13	
Tumor size(cm)						0.741
<= 5	53	5	17	22	9	
> 5	29	2	12	9	6	
Stage						0.008*
I	13	1	5	4	3	
II	24	3	16	4	1	
III	39	2	6	21	10	
IV	6	1	2	2	1	
Recurrence						0.167
With	34	2	15	9	8	
Without	21	4	5	8	4	
NA	27	1	9	14	3	

*$p < 0.05$ was considered statistically significant.
NA: data not available
